# TCR-T Immunotherapy: The Challenges and Solutions

**DOI:** 10.3389/fonc.2021.794183

**Published:** 2022-01-25

**Authors:** Yating Liu, Xin Yan, Fan Zhang, Xiaoxia Zhang, Futian Tang, Zhijian Han, Yumin Li

**Affiliations:** ^1^ Department of Oncology, Lanzhou University Second Hospital, Lanzhou, China; ^2^ Key Laboratory of the Digestive System Tumors of Gansu Province, Lanzhou University Second Hospital, Lanzhou, China

**Keywords:** receptor-engineered T cell, immunotherapy, challenges, solutions, solid tumors

## Abstract

T cell receptor-engineered T cell (TCR-T) therapy is free from the limit of surface antigen expression of the target cells, which is a potential cellular immunotherapy for cancer treatment. Significant advances in the treatment of hematologic malignancies with cellular immunotherapy have aroused the interest of researchers in the treatment of solid tumors. Nevertheless, the overall efficacy of TCR-T cell immunotherapy in solid tumors was not significantly high when compared with hematological malignancies. In this article, we pay attention to the barriers of TCR-T cell immunotherapy for solid tumors, as well as the strategies affecting the efficacy of TCR-T cell immunotherapy. To provide some reference for researchers to better overcome the impact of TCR-T cell efficiency in solid tumors.

## Introduction

Immunotherapy, which is currently changing the concept of cancer treatment, is based on the theory that immune cells can recognize and eliminate cancer cells. Clinical experience has shown that *ex vivo* expanded tumor-infiltrating cells/lymphocytes (TILs) infused into the same patient can produce repeated, even persistent, tumor responses (1). The development of T cell receptor (TCR) isolation technology and genetic engineering programs have enabled patients’ T cells to express TCRs recognizing multiple combinations of specific peptides and human leukocyte antigens (HLA). The resulting TCR-T cells can specifically recognize tumor-associated antigens and effectively eliminate tumor cells.

T lymphocytes with heterogeneous TCRs can recognize HLA-peptide complexes on tumor cells and transmit antigen-stimulating signals through phosphorylation of the immune tyrosine-based activation motif (ITAM), activating the immune effects of T cells to eliminate tumor cells (2). TCR-T technology has undergone four iterations during its development. Initially, isolated tumor antigen-specific T cell subgroups from patients were amplified *in vitro* and re-infused into the same patient. Because these T cell clones are rare with significantly inter-individual variations, their production on an industrial level is difficult. In the second generation of development, tumor antigen-specific T cells were cloned, and their TCR gene sequences were determined. These TCR gene sequences were subsequently transduced into peripheral T cells from patients. This method made it possible to industrialize TCR-T cell production and treatment. In the third generation of development, the therapeutic effects of TCR-T cells were improved by optimizing the affinity of their TCRs to tumor cells. The fourth generation of TCR-T cell treatment is a highly specific cell therapy targeting neoantigens, markedly improving tumor response and patient safety (3). Due to inter-individual variations, however, further studies are required to determine the accessibility of treatment.

**Figure 1:**
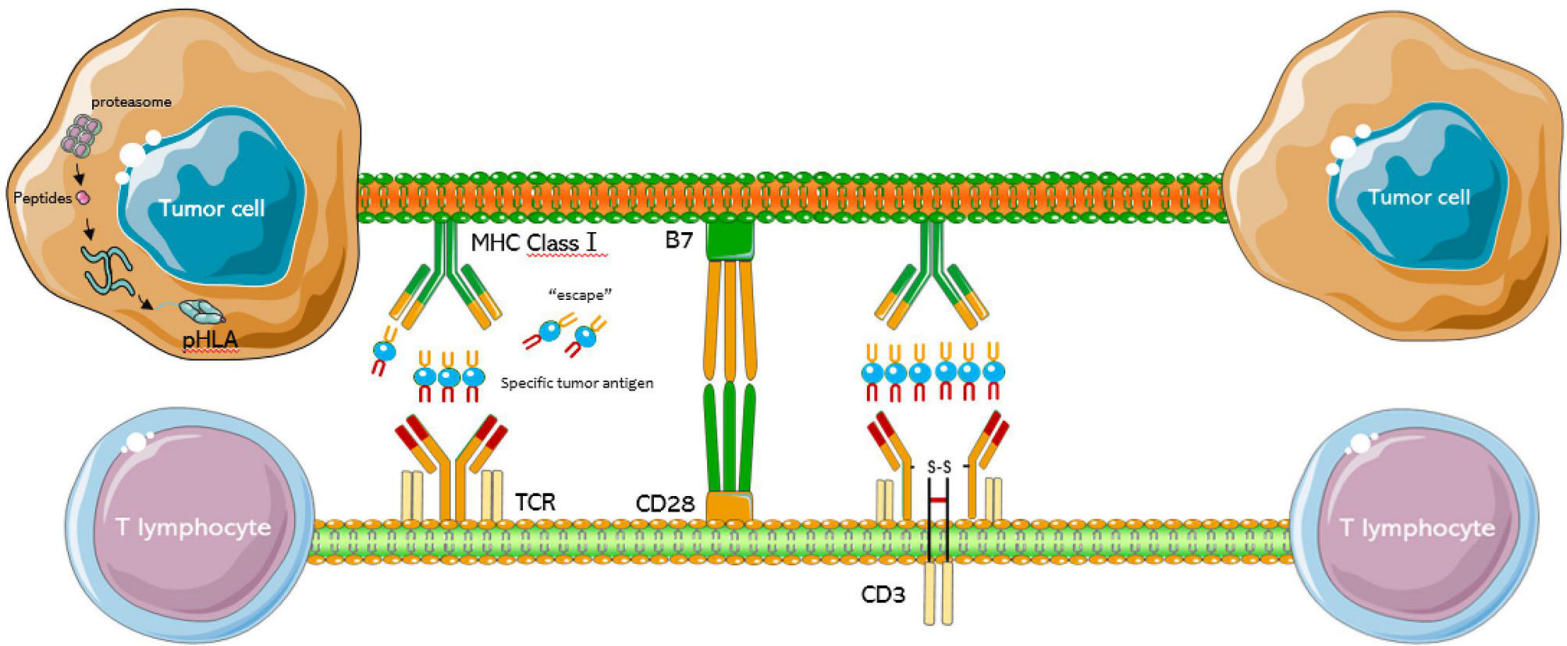
Schematic diagram of the TCR-T cell structure. The TCR complex is a heterodimer consisting of two different peptide chains. The MHC class 1 present intracellular antigenic peptides of cancer cells for recognition by the T cell receptor, and surround by CD28 and B7.

T cell receptor-engineered T cell (TCR-T) therapy is free from the limit of surface antigen expression of the target cells, which is a potential cellular immunotherapy for cancer treatment. Strategic selection of substrate cells can enhance transportation, amplification, durability, and memory function of TCR-T cells, and form a TCR-T cell system with synthetic costimulatory circuit. Further understandings of the barriers to TCR-T therapy treating solid cancer and the solutions will bring benefits to future clinical application of this immunotherapy (**Figure 1**).

## The Challenges in TCR-T Therapy

Insufficient activation of T cells can cause immune escape which can reduce the efficacy of immunotherapy in specific patients (4). TCR-T cells are independent of the patients’ endogenous T cell bank and are not limited by the availability of tumor specific surface proteins. However, constructing a TCR-T cell group that can recognize reliable targets with sufficient affinity and function to eliminate existing tumors and prevent recurrence remains a challenge (5).

### Selection of Target Antigen

Human tumor antigens can be primarily divided into two categories—tumor specific antigens (e.g., neoantigen, and viral antigen) and tumor associated antigens (e.g., cancer/testis (CT) antigen, overexpressed antigen, and differentiation antigen). Although TCR-T cells can target all tumor antigens, the number of targets identified to date with sufficient safety and effectiveness remains limited. The primary consideration in selecting a suitable target antigen for TCR-T cell therapy should be the high specificity of the antigen. Target antigens highly expressed in tumors but at low levels in normal tissue are often selected to limit any potential off-target effects and dose-limiting toxicity resulting from the destruction of normal tissue expressing the target antigen (3). To date, most clinical trials of TCR-T cell therapy have targeted CT antigen and viral antigens, with New York esophageal squamous cell carcinoma-1 (NY-ESO-1) being the most frequently targeted, accounting for 37% of trials to date (6).

At present, overexpressed testicular antigens and differentiation antigens are the most common targets of TCR-based adoptive cell immunotherapy. NY-ESO-1 is a CT antigen with objective response rates of 40-60% in patients with melanoma and synovial sarcoma. Other tumor-specific antigens considered in TCR gene therapy include mutated antigens and neoantigens, most of which are safe targets as they are specifically expressed in tumor cells. Mutated antigens are widely expressed in many tumor types, and neoantigens can be obtained by sequencing due to their individualized characteristics (7). Immune selection pressure may result in the downregulation of expression of target antigens, reducing the efficacy of TCR-T cell therapy (8, 9), especially for specific T lymphocytes. The loss of targeted tumor antigens was shown to result in tumor recurrence even after the infusion of adoptive functional cells (10). The downregulation of target antigens may be overcome by targeting proteins with core functions in tumor survival; infusing multiple T cell clones with different tumor specific TCRs; or infusing T cells targeting two or more tumor antigens (10).

TCRs can only recognize peptide-HLA and kill cancer cells with matching HLA alleles. Because screening of an appropriate HLA match is also necessary. TCR-T cells from non-Chinese individuals cannot be directly applied to Chinese patients. Screening TCRs with the optimal affinity threshold remains difficult. TCRs with high affinity for antigen should be identified to enhance immune responses. However, the affinity should be controlled within limits since T cells will be injured by TCR affinity beyond physiological function. The mechanism of antigen recognition by T cells expressing antigen receptor (TCR) is important for T cell immunity (11), and the ability of T cells to quantitatively respond to antigens expressed by pathogens is an important indicator of T cell response (12). These T cells, however, must remain unresponsive to similar antigens on host tissues (12). The anti-tumor activity of genetically engineered T cells should be enhanced by the transformation process, will alters their affinity. Affinity, however, should be controlled within a certain range, because too low affinity can be toxic to targeted non-tumor tissue, with T cells attacking host nonmalignant tissues expressing tumor related antigens or similar ligands (13). Conversely, affinity should not be too high, as it may lead to abnormal immune activation, increasing the risk of triggering cytokine storms. Moreover, modification of peptide-HLA binding can lead to unpredictable cross reactions against autoantigens, which may cause serious adverse events (14).

The latest generation of TCR-T technology is facing the challenge of effectively identifying antigens. High-throughput sequencing of the immune repertoire (HTS-IR) and computational biology methods including TraCeR and single-cell TCRseq at the population and single-cell levels have been utilized by the company Kite Pharma to reconstruct TCR and identify immunogenic neoantigens. Flow cytometry has been used to select tumor antigen-specific T cells from patients, with TCR genes that recognize these antigens obtained by single-cell technology and introduced into patients’ peripheral T cells for treatment. Such explorations provide new tools for analyzing the diversity and dynamics of T cells. Further bioinformatic progress is required to develop more novel tools.

### Tumor Antigen Heterogeneity and Tumor Immune Escape

In a phase I/II clinical trial (15), a TCR gene targeting MAGE-A3 was transduced into T cells to treat metastatic melanoma, resulting in a response rate of 57% (4/7), with one patient achieving a complete response (CR) for 15 months, and three achieving a partial response (PR). Three other patients, however, developed mental disorders due to brain damage, with two developing severe central nervous system damage and dying of multifocal necrotizing leukoencephalopathy. These adverse effects may have been due to neurotoxicity induced by T cells recognizing antigens cross-reacting with MAGE-A12 in normal brain tissue.

The effect of tumor antigen heterogeneity on the efficacy of TCR-T treatment remains unclear. The expression of cancer-related antigens varies in different cells within tumors, allowing some tumor cells to escape from specific antigen-targeted therapy and leading to therapeutic resistance in some patients receiving immune checkpoint inhibitors (ICIs) (16) and adoptive T cells therapy (17). TCR-T is a T cell product generated by lentivirus transfection, with the resulting cells expressing multiple TCRs against multiple tumor antigens in patient. CRISPR-Cas9 can transfer genes with DNA plasmids or templates derived from polymerase chain reaction, without the need for virus vectors (18). Multiple CRISPR-Cas9 T cell genome engineering has been shown safe and feasible in patients with advanced refractory cancers (19). To reduce the TCR mismatch of two genes encoding endogenous T cell receptor (TCR) chains, TCR α (TRAC) and TCR β (TRBC) were deleted from T cells, the expression of a synthetic tumor specific TCR transgene NY-ESO-1 was enhanced, and the gene encoding programmed cell death protein 1 (PD-1) was removed. Adoptive transfer of engineered T cells into patients can be edited at all three genomic sites to achieve lasting implantation. Of the cells infused into one patient, 30% had undergone editing of two and three genes, with 20% of TCR transgenic T cells in the circulation 4 months later showing the editing of two and three genes. Although chromosome translocation was also detected, the translocation frequency decreased over time. Overall, this study showed that CRISPR-Cas9 gene editing provides a powerful tool for enhancing the natural anticancer activity of human T cells and the feasibility of cancer immunotherapy.

Such optimization may knock out specific genes, resulting in the simultaneous expression of TCR and costimulatory proteins while eliminating inhibitory signals. This can promote the function of TCR-T products by, for example, preventing T cell dysfunction, inhibiting tumor escape, overcoming limited T cell proliferation, and controlling toxicity.

Concerns have arisen about the safety and effectiveness of CRISPR-Cas9 in gene therapy. Humoral, antibody-mediated, cellular and T cell-mediated immunity against *Staphylococcus aureus* (S.aureus; SaCas9) and *Streptococcus pyogenes* (S.pyogenes; SpCas9) have been detected in more than 80% of healthy people; Theoretical analysis showed that activation of these immune responses is accompanied by the signal generation of cytotoxic T lymphocytes (CTL) during pro-inflammatory “dangerous” bacterial infections. The immune system may destroy CRISPR-Cas9 modified genetically engineered cells, making the treatment ineffective, and eliminating infected host cells (20). Therefore, it is reasonable to determine whether the human immune system can generate an anti-Cas9 response. In animal models, anti-Cas9 antibodies have not been found to cause the death of Cas9-expressing cells after gene therapy with non-inflammatory vectors such as AAV. It is unclear whether Cas9 expression can stimulate pre-existing anti-Cas9 immunity, leading to the destruction of transduced cells. If CTLs mediate killing after gene therapy, various strategies can be used to minimize the development and impact of anti-Cas9 T cells.

The discovery of CRISPR-Cas9 has greatly enhanced the effectiveness of gene therapy. The possible clinical application of CRISPR-CAS based treatment technology makes it particularly important to minimize the immunogenicity of gene therapy. Regulatory T cells (Treg) have been reported to promote immune tolerance to gene therapy, and Cas9 specific Treg cells can be enriched from human peripheral blood. Because Cas9 specific Treg cells can promote immune tolerance, treatment with these cells before and during gene therapy can potentially enhance tumor growth. Strict immune monitoring can assess the role of endogenous Cas9 reactive Treg cells during clinical treatment. Therefore, interactions between CRISPR-CAS therapy and Treg cells may allow determination of the relationship between gene therapy and antigen specificity (21).

Tumor immune escape refers to the ability of tumor cells to survive and proliferate *in vivo* by avoiding recognition and attack by the immune system through various mechanisms. Solid tumors are characterized by complex immunosuppressive microenvironment and intrinsic heterogeneity, making a durable TCR-T induced response difficult. Cells and components of the tumor microenvironment (TME) include tumor cells, fibroblasts, immune cells, signaling molecules and extracellular matrices. TME significantly affects tumor diagnosis, patient survival and treatment sensitivity. The TME of different tumor tissues has distinct features. These drawbacks may be overcome by, for example, modifying T cells to release cytokines that can counteract immunosuppressive factors in the TME and combining TME inhibitors (22).

### The Off-Target and Safety Problems in TCR Gene Transfer

Because engineered TCR-T cells cannot distinguish between tumor cells and normal cells expressing target antigens, TCR-T cell immunotherapy may severely damage the corresponding normal tissues. Treatment of metastatic melanoma patients with TCR-T cells targeting MART-1 and MAGE-A3 resulted in fatal cardiotoxicity, perhaps because MART-1 and MAGE-A3 are highly expressed in heart tissue (23, 24). Combinations of chemotherapy and other targeted therapies have been found to result in temporary disease remission and prolong median survival in some patients with disease progression. Inducing TCR-T cell apoptosis or knocking out endogenous TCR may also reduce the adverse effects of TCR-T cell immunotherapy (25).

Off-target events during TCR gene therapy may be caused by self/cross-reactions between heterodimers containing two α and two β chains, which may result in new autoimmune specificity (18). Four different TCRs can form a complex, with two chains derived from exogenous α/β TCRs and the other two from natural/endogenous α/β TCRs. These heterodimeric TCRs can form a receptor with new specificity or a nonfunctional complex. The production of self-reactive T cells from heterodimeric TCRs and the autoimmune manifestations of these cells have been evaluated in a mouse model of TCR gene therapy (26). The autoimmune response induced by the treatment led to the fatal destruction of hematopoietic cell pools, with evidence indicating that this pathological reaction was caused by the formation of mixed TCR dimers (27). Although this TI-associated graft-versus-host disease (GVHD) was similar to transfusion related graft-versus-host disease (TA-GVHD), the occurrence of TI-GVHD was significantly reduced when mouse TCR transduced T cells were cultured *in vitro* for 10 days. This observation is in good agreement with the results of early adoptive cell metastasis, which showed that the function of “older” T cells was greatly weakened *in vivo*. Alternatively, the proportion of CD4 + T cells in most cell grafts injected into patients is relatively low, with CD4 + T cells playing an important role in the occurrence of TI-GVHD. TCR dimer dependent mixed toxicity may also become a safety problem in clinical applications. Transfer of a TCR gene to human T cells can result in the formation of mixed TCR dimers, which can induce a specific response *in vitro*. These findings emphasize the importance of evaluating and implementing techniques that can prevent TI-GVHD.

Methods, primarily molecular methods, have been developed to improve the expression level of introduced TCR. These methods were designed to provide better match/association between the α/β chains of exogeneous TCR (28). For example, introducing partial/whole genes from the mouse constant region into human TCR can increase TCR transduction levels.

**Figure 2:**
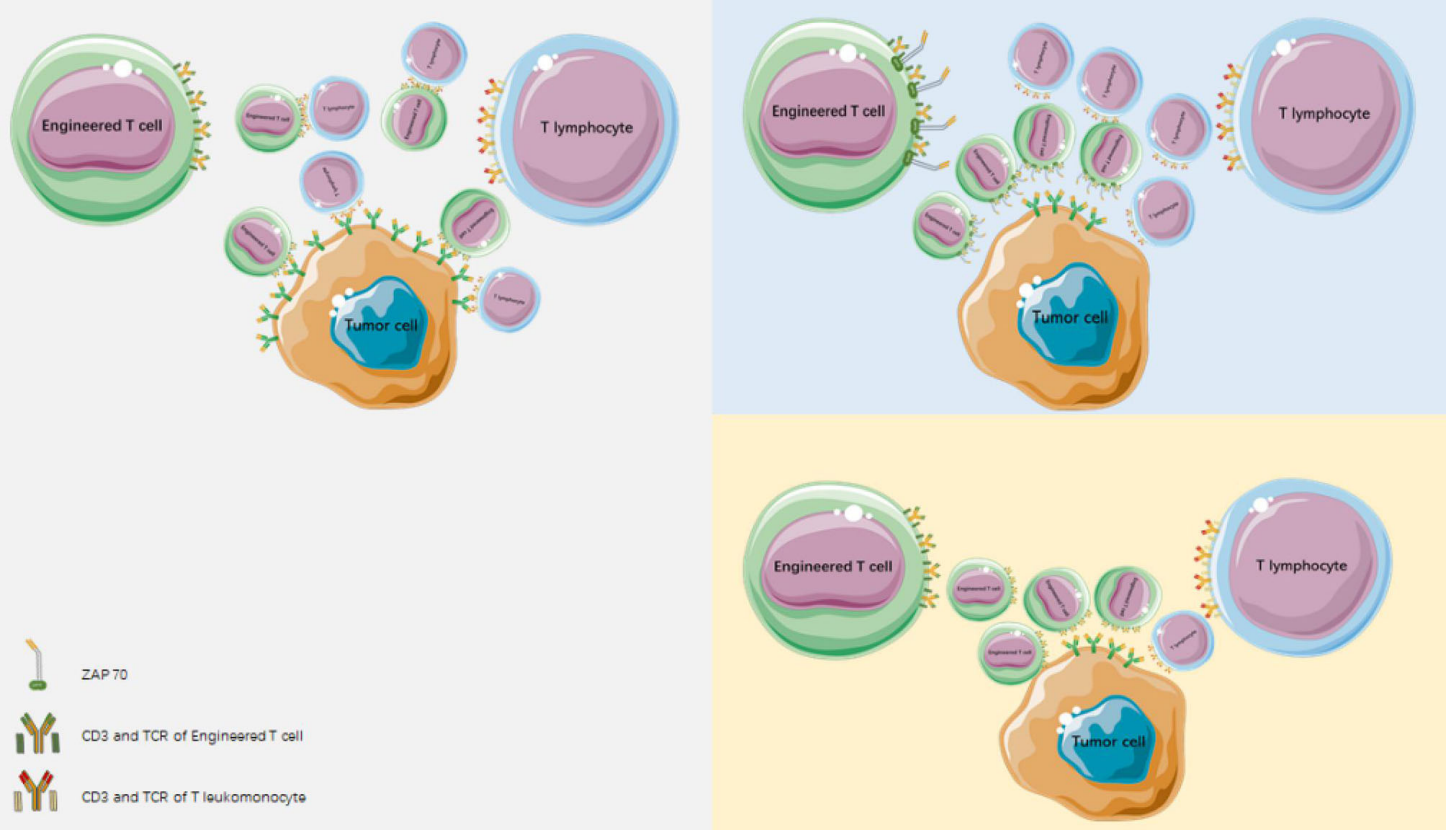
Reducing mismatches between endogenous and heterogeneous TCR and reducing competition for CD3 molecules can mediate an increase in surface expression of modified TCR cells.

Other matching optimization methods include introducing an extra disulfide bond into the TCR constant region (29, 30), the use of single-stranded TCR (31, 32), and the application of TCR/CD3 fusion products (33). Because the α/β and γ/δ chains of TCR cannot match each other (25), the use of α/β TCR-transduced T cells is an alternative (34). Alternatively, endogenous TCR can be silenced by cotransferring endogenous TCR siRNA/shRNA or TCR chain-specific zinc finger nuclease (35). The CD3 molecules associated with modified and endogenous TCR compete with each other, significantly reducing the expression of heterodimer on the surface of modified TCR cells and impairing T-cell function. Retrovirus vectors and siRNA have been used to silence endogenous TCR gene expression and optimize tumor antigen-specific TCR. The transduction of lymphocytes with a relatively low number of provirus particles, resulted in the effective expression of the introduced TCR, reduced the expression of endogenous TCR, and enhanced the antigen-specific lysis of target cells. Specific siRNAs acting on the TCR constant region which increased and optimized cell surface expression of MAGE-A4-specific TCR. Reducing mismatches between endogenous and exogeneous TCRs and their competition for CD3 molecules can increase the surface expression of modified TCR cells (36) (**Figure 2**).

### T Cell Unresponsiveness and Exhaustion

The efficacy of treatment with chimeric antigen receptor-T (CAR-T) cells and T cell receptor-engineered T cells is highly dependent on the functional activity of these T cells. However, the mechanisms underlying T cell exhaustion remain unclear. T cell unresponsiveness represents a low-reactive state of T cells, triggered by over activation of TCR and strong co-inhibition, either through CD28 molecular signals or limited co-stimulation. Repeated T cell activation during chronic infection or tumor progression can lead to T cell exhaustion. During the clearing of acute infection, a population of activated T cells differentiates into high-functioning memory T cells; whereas, during chronic infection or in the TME, sustaining the activation of T cells can result in a dysfunctional phenotype, which is characterized by poor effector functions and expression of inhibitory receptors (37). Dysfunctional T cells cannot produce interleukin (IL)-2 and gradually lose their proliferation and *in vitro* killing abilities, subsequently becoming unable to secrete tumor necrosis factor (TNF)-α. In the final stage, interferon-γ production is partially or completely impaired, eventually leading to physical loss. The decline in effector function is accompanied by a gradual loss of CD4+ T cells and increased expression of inhibitory receptors, including CD160, CD244, CTLA4, LAG-3, PD1, TIGIT, and TIM3 (38, 39).

The depletion of early memory T cells and other T cells can alter the effectiveness of genetically engineered T cell therapy (40). Dysfunctional T cells are the main proliferating immune cells in tumors, with the intensity of dysfunction being associated with tumor reactivity (41). Depleted CD8 + T cells can include progenitor cell depleted T cell subsets, which can remain versatile and eventually differentiate into depleted T cells after a long period of time (42, 43). CD8 + T cells before failure can be evaluated by the expression of the cell surface inhibitory receptor PD-1, the chemokine receptor CXCR5 and the soluble factor TCF-1 (44, 45).

T cell dysfunction and exhaustion are important drawbacks to engineered T cell therapy. The dysfunction and exhaustion phenotypes of endogenous T cells are derived from the surrounding TME (46, 47) and are induced towards terminal differentiation. PD-1 upregulation in the TME significantly inhibits T cell function, so engineered T cells generated from impaired T cells may have lower efficacy against hematologic malignancies and solid tumors (48–50). In addition, endogenous TCR of the T cell may have a persistent negative impact on engineered T cells. Finally, signals from transformed T cells may increase cell differentiation and exhaustion (51, 52).

In summary, the key to enhancing the anti-tumor function of engineered T cells is adjusting tumor-associated T cell dysfunction and exhaustion. Currently, there are three main strategies to restore T cell pool: replacement, reprogramming and exhausted cell recovery.

1) Replacement involves the physical removal of dysfunctional cells from the circulation to ensure the homeostatic proliferation of effector and memory T cells. One possible approach is to target dysfunctional T cells and promote their selective apoptosis. An engineered peptide was used to interfere with FOXO4/p53, leading to the apoptosis of senescent fibroblasts (53). It is not clear whether this approach is also applicable to dysfunctional T cells. However, homeostatic proliferation, as in autologous stem cell transplantation (ASCT), has resulted in the successful reconstruction of functionally naive, memory, and effector T cell pools in autoimmune diseases and hematologic malignancies (54–57). In addition, hematopoietic stem cells isolated from umbilical cord blood have been utilized to rebuild immune systems and treat hematological diseases, conditions that may allow for the homeostatic proliferation of effector T cells (58–60).

2) Reprogramming is a practical approach to rescue T cells from a state of dysfunction and exhaustion. It involves the redifferentiation of induced pluripotent stem cells originally derived from T cells (T-iPSCs) or their dedifferentiation within their own lineages (61–63). Although the generation of T cells from human embryonic stem cells and iPSCs is feasible, the TCRs generated by seemingly random VDJ gene rearrangements remain unpredictable. Human iPSCs-derived T cells transfected with tumor antigen-specific engineered TCRs and CARs were found to infiltrate solid tumor tissue in a xenograft model and delay tumor progression (64). Moreover, CTL regenerated from iPSCs, including WT1 antigen specific CTLs, showed therapeutic effects in xenograft leukemia models (65). These cells also showed a strong therapeutic effect in an orthotopic xenotransplantation model using a renal cell carcinoma cell line. This method was expanded by transducing HLA haplotype homozygous iPSCs with the WT1 specific TCRα/β gene, a method that has been tested clinically. Moreover, the feasibility of this anti-solid tumor strategy was shown in a patient-derived renal cell carcinoma xenotransplantation model, in which regenerated antigen-specific CTLs inhibited tumor growth.

**Figure 3:**
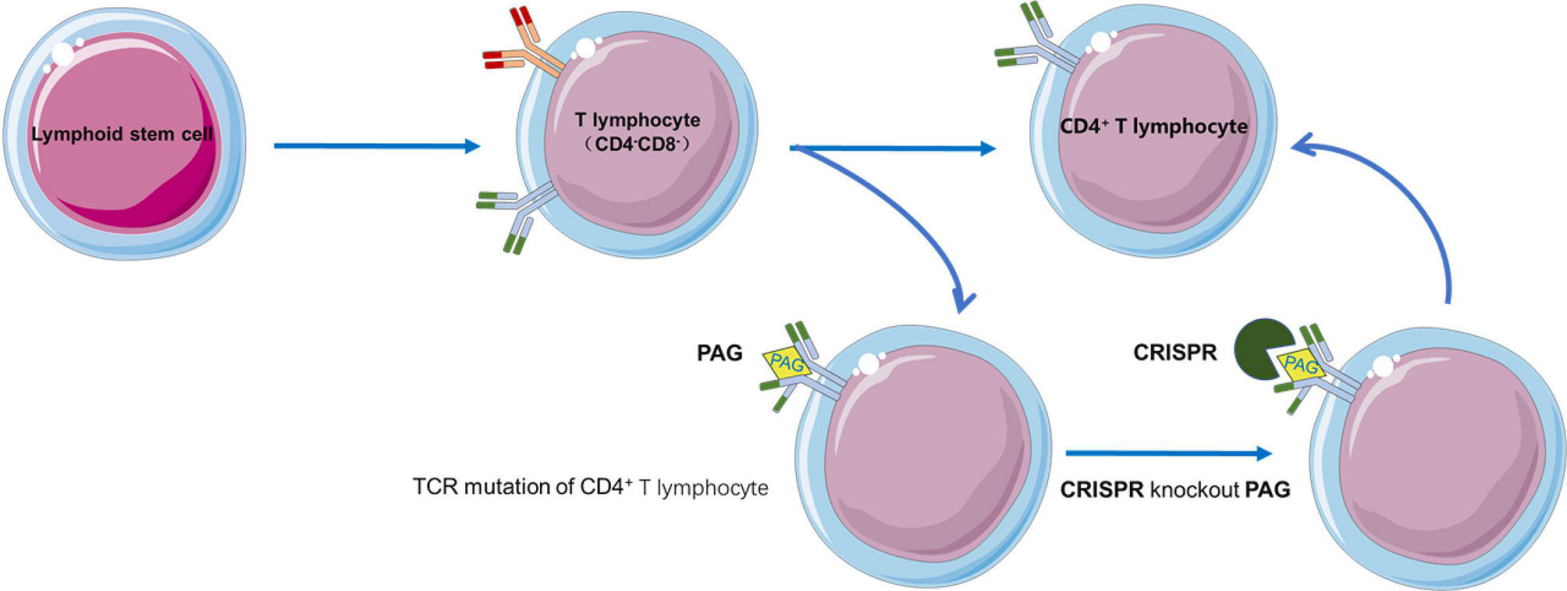
RAG2 knockout by CRISPR-based genome editing in T-iPSCs prevents the additional TCR rearrangement.

The DP stage of iPSCs differentiation into T cells is frequently characterized by additional T cell receptor changes, which may alter antigen specificity; RAG2 knockout by CRISPR-based genome editing of T-iPSCs was found to inhibit additional TCRα rearrangement (66). The combined transduction of HLA matched allogeneic iPSCs and TCRs is expected to promote allogeneic adoptive T cell immunotherapy. Moreover, reprogramming can restore telomere length by increasing telomerase activity, prolonging cell life span by preventing telomere-dependent cell exhaustion, which may reverse T cell exhaustion (67, 68) (**Figure 3**).

3) The recovery method aims to restore and maintain the thymus environment by bioengineering thymic organoid substances, growth-promoting factors, and cytokines (e.g., IL-21), and to further reverse thymus degeneration. IL-21, a type of thymus-stimulating cytokine that can trigger new thymus formation in aged mice, has shown significant immune storage function and promotes peripheral T cell pool regeneration (69, 70).

Similarly, in a mouse model of severe combined immunodeficiency, injection of allogeneic hematopoietic cells into the reconstructed thymus restored functional T cell development (71). Preclinical studies have shown that the production of thymic organoids from acellular matrix is an effective way to restore T cell and adaptive immune system function. However, donor-specific immune tolerance, complex thymic extracellular matrix (ECM) regeneration, thymic epithelial cell support, and T cell maturation remain major challenges (72, 73).

### Toxicity Cause by Cytokine Storms

The toxic reactions related to genetically engineered T cell immunotherapy can be divided into two types, autoimmune toxicity and cytokine related toxicity. Autoimmune toxicity, also called targeted and non-tumor toxicity, results from the antigen-specific attack on nonmalignant host tissue expressing the targeted tumor related antigen. Autoimmune toxicity can occur after the administration of immune checkpoint inhibitors (12–14) and the infusion of genetically engineered T cells (14, 23, 74). Cytokine related toxicity, also known as cytokine release syndrome (CRS), results from high-level immune activation and is a type of non-antigen-specific toxicity.

The incidence and severity of CRS associated with adoptive T cell therapy for tumors were shown to increase in patients with a large tumor load, increases that may be associated with higher levels of T cell activation. Similar to CRS related to monoclonal antibody therapy, CRS related to adoptive T cell therapy is closely associated with increases in IL-6 and TNF-α levels, accompanied by increases in IL-2, granulocyte macrophage colony stimulating factor (GM-CSF), IL-5, IL-8 and IL-10 (75–78). IL-6, the central mediator of CRS toxicity (79), has been shown to play important roles in the activation, expansion, survival and polarization of T cells (80), and to promote the expression of T cell adhesion factor (81). In addition, IL-6 has also been found to regulate the surface expression of Fas receptor by upregulating anti-apoptotic factors through STAT3, thus inhibiting T cell apoptosis (82, 83).

IL-6 has also been shown to be involved in the accumulation of cytokines in bone marrow mesenchymal stem cells present in tumors (84). In addition, IL-6 plays an important role in acute immune response. When stimulated by local inflammation, IL-6 can promote the production of acute phase proteins by acting on the liver (85). IL-6 is an important factor affecting the homeostasis of hepatocytes, hematopoietic progenitor cells, and the cardiovascular, endocrine and nervous systems (79). Moreover, many clinical trials have shown that high expression of IL-6 is related to CRS, initiating the pro-inflammatory signal cascade (86).

The release of cytokines by cytotoxic T cells resulting in T cell receptor activation and abnormal macrophage activation may eventually lead to hemophagocytic lymphohistiocytosis (HLH) (87). The main characteristics of HLH are improper immune activation and cytokine release. Primary HLH is caused by mutations in genes involved in cytolytic granule exocytosis, reducing natural killer (NK) function and allowing macrophages to activate spontaneously in response to minimal triggering (88). Secondary HLH is caused by infections, malignant tumors and autoimmune diseases. The symptoms of HLH in some patients were relieved after treatment with IL-6 receptor blockers. Host factors may play an important role in immunotherapy for individuals prone to severe CRS, although additional studies are needed to determine whether heredity is related to this syndrome (87).

## Other Improved Methods for Enhancing the Function of Engineered T Cells

Although some patients have been found to benefit from TCR-T therapy, efforts are required to improve the amplification/durability of T cells *in vivo* to avoid rapid loss of effector function. This may improve response rates in patients with high tumor burden and reduce the need for large amounts of TCR-T cells. Because TCR insertion alone may be insufficient for potent anti-tumor responses (89), other features of co-stimulating receptors and TRC “substrate” cells may provide potential methods to overcome these obstacles and to improve clinical outcomes.

### Increased Structural Affinity Can Enhance Anti-Tumor Function

Most clinical studies of TCR-T cell immunotherapy to date have focused on melanoma. Targets have included melanoma associated antigen recognized by T-cells (MART)-1, P-glycoprotein 100 (P-gp100), NY-ESO-1, MART-A3, and p53. To determine whether reconstructed T lymphocytes are effective in the treatment of melanoma, the genes encoding the α and β chains of MART-1 specific TCR were transfected into peripheral blood mononuclear cells (PBMCs) with retroviral vectors, and the recombinant T cells were used to treat 17 patients with metastatic melanoma (90). In this phase I clinical trial, two (12%) patients achieved PR for over 20 months, and the other 15 (88%) patients showed ≥10% increases in peripheral blood T cell counts after 2 months of treatment, with no treatment-related adverse events.

In another trial, patients with metastatic melanoma were treated with TCR-T cells expressing MART-1 or P-gp100 and possessing high TCR activity (91). PR was observed in 30% (6/20) and 13% (2/16) of patients treated with TCR-T cells expressing MART-1 and P-gp100, respectively, with one patient in the latter group achieving CR. However, secondary damage was observed in normal melanocytes of the skin and eyes, and tertiary damage in ears. These results indicate that engineered T cells differ in their ability to distinguish between normal and tumor cells, leading to varying therapeutic effects. Therefore, in addition to high affinity, thus ability to distinguish should be considered when designing TCR. This a technical bottleneck has restricted TCR-T development. Specifically, improvements in affinity can increase the risk of TCR attack of off-target sites, resulting in off-target toxicity, and targeting similar antigenic epitopes in healthy tissue can damage the latter. To date, however, only 1% to 2% of tumor mutations in new antigens can be combined with MHC molecules to be identified by T cells, indicating that new antigen-specific T cells have a limited ability to recognize tumor tissue with a low mutation load (92).

Because T-cells administered anywhere in the body have the potential to be actively transported throughout of the body, it is important to select the right target. Most potential targets are not tumor-specific and are expressed in lower concentrations in healthy tissues, increasing the risk of off-target effects. Even if highly expressed or specific tumor targets can be identified, they are not evenly expressed throughout the tumor due to tumor heterogeneity. T-cell therapy targeting antigens not present in all tumor cells may lead to the selective growth of target-negative tumor cells (93). Therefore, the construction of TCR-T cells with common new antigens, new antigens covering most tumor subclones, and driving mutations of new antigens is expected to improve the therapeutic effect of new antigen TCR-T, benefitting more patients.

Increasing the structural affinity of TCR may enhance their anti-tumor activity. 1) The introduction of selective modifications to the CDR3 region of TCR α and β chains has proven critical for antigen recognition and binding (94). 2) Pairing and codon optimization, thus increasing protein expression, may enhance the antigen-specific responses of T cells. 3) Reducing the glycosylation of TCR improves its functional affinity and prevents the internalization of transduced TCR. 4) Modifying three transmembrane residues of the TCR α chain to hydrophobic amino acids can enhance the anti-tumor functional affinity of T cells, as well as increasing the stability and level of expression of TCR in these transduced T cells (95). 5) The design of gene expression box may also affect TCR expression, and the application of P2A or IRES elements linking α and β chains has been shown to increase TCR expression levels and reduce the risk of inducing autoimmune pathological changes (96).

In addition to the T cell-specific engineering of TCR transgenes, several genetic approaches have been utilized to further induce or amplify important T-cell functions (e.g., co-stimulation, cytokine secretion, and expression of chemokine receptors and homing factors) (97). For example, IL-12 administration into tumor mouse models can promote tumor regression and improve host survival (97), although it also enhanced toxicity. Inducing engineered T cells to produce IL-12 *in vivo* through retroviral vectors has been shown to enhance anti-tumor activity against melanoma in B16 mice (98). Combining T cells with cytokine adjuvant nanoparticles can result in the local production or delivery of cytokines with reduced toxicity (66). Subtypes of the transduced T cells are also important, with recent studies showing the favorable performance of various lymphocyte subsets, including memory T cells, primary T cells, memory stem cells and central memory T cells (97–99).

### The Necessity of Stromal Cell Selection

In preclinical models, the naïve, central memory and stem cell memory subsets of CD8+ T lymphocytes mediated the durability and anti-tumor effects of adoptive T cell therapy (100). These findings were further confirmed by a clinical trial in patients with acute myeloid leukemia using WT-1 specific TCRs to transduce donor EBV-specific CD8+ T cells (101). The transduced TCR-T cells maintained the expression of costimulatory receptors, with lower levels of expression of inhibitory receptors, which may mediate the stability of TCR-T frequency and durability observed in patients. However, not all patients benefited from EBV-specific substrate cells, especially those with high tumor burden and activation-induced T cell death. This suggests that alternative approaches are necessary.

In addition to TCR signaling, T cell function is regulated both positively and negatively ways. For example, the TME can induce immunosuppression, and transforming growth factor-β (TGF-β) can inhibit T cell proliferation and function (102). Tumor cells expressing TGF-β may escape from apoptosis (103, 104). TGF-β-induced inhibition may be reduced by introducing a truncated, dominant negative TGF-β receptor into genetically engineered T cells (105).

### The Combination of CD4+ T Cells Can Enhance the Efficacy of TCR-T Therapy

Current TCR-based immunotherapy mainly utilizes CD8+ T cells that recognize tumor antigen presented by class I HLA (106). Cotransfected CD4+T cells can enhance antitumor effects by promoting the proliferation and survival of tumor-killing CD8+T cells, as shown in murine leukemia models (107) and in CD19-directed CAR therapy (108). Tumor-specific class II-restricted CD4+T cells promote class I-restricted CD8+T cell proliferation, survival, and effector function, partially by producing IL-2 and promoting dendritic cell-mediated activation to expand the range of immune responses (epitope diffusion) (109). CD4+T cells expressing class II HLA-restricted TCR show direct cytolytic activity against metastatic melanoma and antitumor effect against human cholangiocarcinoma (110, 111). However, class II expression is rare in solid tumors (112) and it is difficult distinguish class I and II restricted TCRs of the same tumor antigen.

Viral and neoantigen-specific class I TCRs have sufficient affinity to bind to CD4+ and CD8+T cells, but thymus selection results in TCRs that rarely recognize overexpressed autoantigens. Identification of CD8-independent class I HLA-restricted TCRs requires extensive screening but is not always successful for each target. Co-expression of the α and β chains of CD8 with TCR, an alternative for binding CD4+ and CD8+T cells, may be applied to any class of restricted TCR (113). In some preclinical models, tumor specific CD4+ T cells support CD8+ T cell proliferation and function (114). However, participation of CD4+ T cells alone may be insufficient in the complex TME.

Obtaining HLA class I restricted gene modified CD4+ T cells was shown to be feasible after cloning or spontaneous transfer of the CTR gene from targeted specific CD8+ T cells (115). WT1 siTCR/CD4+ T cells can produce Th1 cytokines and enhance WT1 reactive CTL function mediated by WT1 siTCR/CD8+ T cells. Moreover, administration of WT1 siTCR/CD4+ T cells not only enhances the killing effect of anti-leukemia cells, but enhances the proliferation and differentiation of WT1 siTCR/CD8+ T cells during the process of recognizing leukemia cells *in vivo*. In addition, WT1 siTCR/CD4+ T cells express the chemokine CXCL12 and its receptor CXCR4. CD4+ T cells expressing CXCR4 can enter the bone marrow through the CXCR4-CXCL12 axis. Therefore, intravenously administered WT1 siTCR/CD4+ T cells can not only be transported to peripheral blood, but also enter the patient’s bone marrow. Administration of WT1 siTCR/CD4+ T cells was also found to increase the accumulation of WT1 siTCR/CD8+ T cells around leukemia cells, thus enhancing their inhibitory effect. In addition, the presence of WT1 siTCR/CD4+ T cells can prolong the survival of functional WT1 siTCR/CD8+ T cells *in vivo*. These findings indicate that leukemia can be most effectively inhibited by simultaneous administration of WT1 siTCR/CD4+ T cells and WT1 siTCR/CD8 + T cells, and that this enhanced anti-leukemia effect is significant. That is, target specific CD4+ T cells, can enhance the duration of the anti-leukemia effect of adoptive gene modified CD8 + T cells.

### Independent Activation of Costimulatory Receptors Is Essential for Improvement of TCR-T Therapy

The incorporation of costimulatory domains (most notably CD28 and 41BB, and also ICOS and OX40) into intracellular CAR-T signal transduction domains has been shown essential for CAR-T cell function. CD19 CAR-T cells have shown antitumor effects against hematological malignancies (116). The combination of TCR and MHC initiates TCR signaling, leading to the formation of immune synapses between T cells and antigen-preventing cells (APCs) (117). These interactions between T cells and APCs can simultaneously trigger the binding of costimulatory signal receptors (such as CD28) and lead to the recruitment of CD8 or CD4 co-receptors, which bind to conserved regions in MHC class I or MHC class II complexes, respectively (118). The intracellular domains of CD4 and CD8 can recruit the tyrosine kinase LCK, a member of the SRC family, to bound TCR:pMHC. TCR:pMHC interaction with TCR promotes the LCK mediated phosphorylation of the ITAM in the cytoplasmic domain of the CD3 subunit (118). This phosphorylation can lead to the recruitment of the protein tyrosine kinase ZAP70 to TCR and promote the activation of ZAP70 by LCK. The early T cell activation model hypothesized that the initiation of TCR signaling involved and required the full activation of LCK (119). Subsequent research, however, showed that a large percentage (up to 40%) of LCK had catalytic activity in resting T cells, with recent studies indicating that the active pool of LCK in resting T cells may be smaller than originally estimated (120). In addition, full activation of T cells was found to require the matching of tumor ligands to independently triggered costimulatory receptors, while overcoming inhibitory receptor signals through highly expressed ligands in the TME (121).

**Figure 4:**
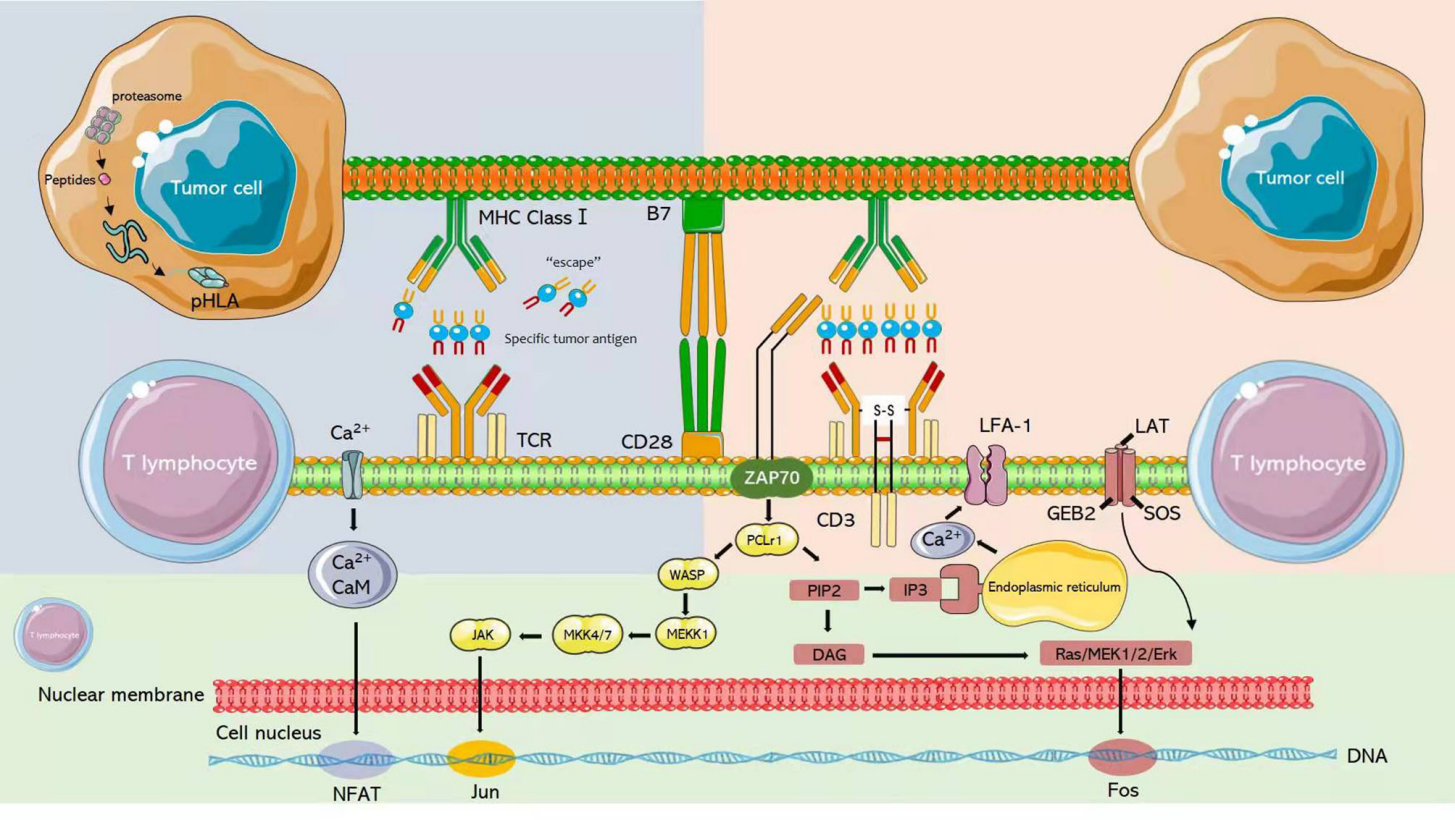
After T cells interact with MHC through TCR, several proteins will be recruited to the plasma membrane to participate in signal transduction.

The interaction of T cells with the MHC through TCR results in the recruitment of several proteins to the plasma membrane, where they participate in signal transduction (**Figure 4**). The initial TCR signal continuously induces phospholipase C through p38- γ1(PLC-γ1) and vitamin D receptor (VDR), both of which are classical TCR signaling pathways necessary for T cell activation (122). PLC-γ1 membrane phosphatidylinositol diphosphate (PIP2) was found to composed into inositol triphosphate (IP3) and diacylglycerol (DAG). IP3, in turn, interacts with the endoplasmic reticulum receptor to upregulate the level of intracellular Ca^2 +^, activating the binding protein calmodulin and nuclear factor (NFAT) protein 2 of T cells. In addition, DAG activates the Ras extracellular regulated kinase (ERK) pathway, ultimately activating nuclear factor (FOS) protein (123). These interacting signaling pathways induce the activation of T cells and the release of large numbers of cytokines and chemokines (124).

A recent preclinical study showed that enhanced costimulatory signals can promote TCR-T cell proliferation, cytotoxicity, and cytokine production (125). Specific structures tailored for optimal TCR-T cell activation have not been utilized to date in clinical studies, as several factors require consideration. First, the selection of costimulatory domains will affect mitochondrial biogenesis and overall cell metabolism, leading to the differentiation of effector molecules and central memory T cells and changes in T cell dynamics (126, 127). Second, the location of the costimulatory domain in the immune synapse will affect the design. The so-called switch receptors (e.g., CD20R/CD28 and PD1/CD28) have shown great efficacy, both *in vitro* and in mouse models, by inhibitory to stimulatory signals (128, 129). However, this approach relies on additional ligand interactions in the TME rather than TCR:pHLA interaction. Insertion of these structures alongside the TCR requires two separate carriers or a large carrier, limiting the efficiency of the transduction. Third, target cell abundance and TCR affinity are also key factors. An increase in the number of target cells, will result in the amplification of TCR signals by TCR:pHLA interactions. This, in turn, may lead to a harmful cytokine storm or T cell tolerance affecting safety and antitumor efficacy (130). In addition, the efficacy of CAR-T cells (which have intrinsic costimulatory properties) is limited in solid tumors (116). These findings suggest that TCR-T therapy requires engineering based on increased costimulatory signaling.

Promising approaches include removing signals that suppress TCR activation, increasing tumor localization and penetrance, and altering T cell metabolism.

The number of preclinical constructs designed to enhance downstream T cell function triggered by TCR highlights the need for unbiased systematic library screening to reveal the potential synergistic effects among antigen targets, transgenic TCR, and increased engineered-T cell adaptation.

## Prospect and Summary of Tcr-T Cell Immunotherapy

TCR-T cell therapy is a powerful immunotherapy to treat tumor. Its complexity makes it challenging in preclinical optimization and in clinical trials. This review indicates that TCR-T therapy can be further improved to reach its full potential (131). Optimizations include systemic selection of TCR-T target antigen, the influence of tumor antigen heterogeneity, and safety problems in TCR gene transfer. Meanwhile, with the development in concepts and technology have resulted in new gene engineering approaches to enhance TCR-T cell function and optimize anti-tumor immune responses (132). This optimization is a complex interdisciplinary issue that integrates immune-oncology, tumor biology and genetic engineering.

Clinical application of TCR-T cell therapy remains challenging. Complicated genetic engineering involving the knockout of multiple genes and individualized treatment can enhance both the safety and efficacy of these treatments. We believe that addressing these issues and applying them to the development of TCR-T cell products will promote TCR-T cell to become an important component of anticancer therapy.

## Author Contributions

YTL: Writing-Original draft preparation, Investigation, and figure preparation. XY: Investigation and figure preparation. XZ: Investigation. FZ: Investigation FT: Investigation. ZH: Figure preparation. YML: Conceptualization, Methodology, Supervision. All authors contributed to the article and approved the submitted version.

## Funding

This work was supported by Special Research Project of Lanzhou University Serving the Economic and Social Development of Gansu Province (054000282), the Natural Science Foundation of Gansu Province, China (Grant No.21JR1RA121) and Fundamental Research Funds for the Central Universities (lzujbky-2020-kb14).

## Conflict of Interest

The authors declare that the research was conducted in the absence of any commercial or financial relationships that could be construed as a potential conflict of interest.

## Publisher’s Note

All claims expressed in this article are solely those of the authors and do not necessarily represent those of their affiliated organizations, or those of the publisher, the editors and the reviewers. Any product that may be evaluated in this article, or claim that may be made by its manufacturer, is not guaranteed or endorsed by the publisher.

## Abbreviations

ASCT, autologous stem cell transplantation; APCs, antigen-preventing cells; CAR-T, chimeric antigen receptor-T; CR, complete response; CRS, cytokine release syndrome; CT antigen, cancer/testis antigen; CTL, cytotoxic T lymphocytes; DAG, diacylglycerol; ECM, extracellular matrix; ERK, extracellular regulated kinase; GM-CSF, granulocyte macrophage colony stimulating factor; GVHD, graft-versus-host disease; HLA, human leukocyte antigens; HLH, hemophagocytic lymphohistiocytosis; HTS-IR, High-throughput sequencing of the immune repertoire; ICIs, immune checkpoint inhibitors; IL, interleukin; IP3, inositol triphosphate; ITAM, immune tyrosine-based activation motif; MART, melanoma associated antigen recognized by T-cells; NK, natural killer; NY-ESO-1, New York esophageal squamous cell carcinoma-1; PBMCs, peripheral blood mononuclear cells; PD-1, programmed cell death protein 1; P-gp100, P-glycoprotein 100; pHLA, peptide-HLA; PIP2, phosphatidylinositol diphosphate; PLC-γ1, phospholipase C through p38- γ1; PR, partial response; SaCas9, Staphylococcus aureus; SpCas9, Streptococcus pyogenes; TA-GVHD, transfusion related graft-versus-host disease; TCR, T cell receptor; TCR-T, T cell receptor-engineered T cell; TGF-β, transforming growth factor-β; TI-GVHD, TI-associated graft-versus-host disease; TILs, tumor-infiltrating cells/lymphocytes; T-IPSCs, induced pluripotent stem cells originally derived from T cells; TME, tumor microenvironment; TNF, tumor necrosis factor; TRAC, TCR α chain; TRBC, TCR β chain; Treg, Regulatory T cells; VDR, vitamin D receptor.
